# NMR resonance assignments of the archaeal ribosomal protein L7Ae in the apo form and bound to a 25 nt RNA

**DOI:** 10.1007/s12104-014-9569-8

**Published:** 2014-07-17

**Authors:** Thomas Moschen, Christoph Wunderlich, Christoph Kreutz, Martin Tollinger

**Affiliations:** Center for Molecular Biosciences Innsbruck (CMBI), Institute of Organic Chemistry, University of Innsbruck, Innrain 80/82, 6020 Innsbruck, Austria

**Keywords:** NMR, Assignment, L7Ae, Kink-turn, Ribosomal protein

## Abstract

The archaeal protein L7Ae forms part of a protein complex in the ribosome that specifically recognizes and binds to kink-turn RNA. In this complex, L7Ae directly interacts with the oligonucleotide and creates a functional arrangement for site-specific 2′-*O*-methylation. We report the solution NMR backbone assignment of *Methanocaldococcus jannaschii* L7Ae (117 residues, 12.7 kDa) in the ligand-free state and when bound to a 25 nucleotide C/D box kink-turn mimic RNA.

## Biological context

The archaeal ribosomal protein L7Ae of *Methanocaldococcus jannaschii* belongs to a family of proteins that recognize and bind kink-turn motifs in ribosomal and box C/D as well as box H/ACA RNAs. In the crystal structure of L7Ae bound to a kink-turn derived from an archaeal box H/ACA sRNA the protein folds into a compact globular domain comprising a four-stranded central β-sheet that is surrounded by a total of five α-helices and a short 3_10_-helix (Suryadi et al. 2005). L7Ae interacts with RNA by docking into its major groove, which stabilizes the kink-turn conformation of the oligonucleotide and creates the functional three-dimensional arrangement that is required for site-specific 2′-*O*-methylation by fibrillarin (Huang and Lilley 2013). Crystallographic analysis of ligand-free L7Ae from *M. jannaschii* showed that only minimal conformational differences between ligand-free and RNA-bound L7Ae exist (Hamma and Ferré-D’Amaré 2004).

Here we report the solution NMR backbone and partial side chain assignment of the *M. jannaschii* protein L7Ae (117 residues, 12.7 kDa) in the ligand-free state and when bound to a 25 nucleotide C/D box kink-turn mimic RNA. Our assignments lay the foundation for NMR studies of protein dynamics and binding interactions between L7Ae and RNA.

## Methods and experiments

### Protein expression and purification

A pET-28a vector encoding N-terminal His_6_-tagged L7Ae protein carrying kanamycin resistance was kindly provided by Keith Gagnon (University of Texas, Southwestern Medical Center, Dallas, TX). Expression of ^15^N/^13^C labeled samples was carried out in M9 minimal medium (containing 25 µg/ml kanamycin) with ^15^NH_4_Cl and ^13^C_6_-glucose as sole nitrogen and carbon sources, respectively, using *E. coli* BL21 cells. Overexpression was induced with 0.5 mM IPTG. Because L7Ae is a nucleic acid binding protein, after harvesting the cells by centrifugation at 4,000 rpm, 4 °C, the cell pellet was re-suspended in denaturing buffer A (20 mM TrisHCl pH 7.5, 250 mM NaCl, 10 mM imidazole, 6 M urea). The cells were lysed by sonication and the lysate was passed through a 45 µm filter before loading on a 5 ml HisTrap excel preloaded Ni-column (GE Healthcare). The protein was eluted within a 20 ml gradient from 0 to 100 % buffer B (same as buffer A but with 500 mM imidazole). Subsequently, the fractions containing L7Ae were loaded onto a size exclusion column (320 ml Superdex 75 26/600, GE Healthcare) and eluted with 50 mM potassium phosphate and 25 mM NaCl (KP buffer). L7Ae was concentrated to 1 ml using Amicon Ultra Centrifugal Filters with 3 kDa cutoff (Millipore) and the His-tag was cleaved with thrombin (5 U, Merck Millipore) overnight at room temperature. The protein was further purified by size exclusion chromatography (320 ml Superdex 75 26/600 column, GE Healthcare) with KP buffer. Fractions of pure L7Ae eluting at ca. 198 ml were concentrated to a final protein concentration of approximately 1 mM using Amicon Ultra Centrifugal Filters with 3 kDa cutoff (Millipore). The buffer was exchanged to 10 mM sodium cacodylate, pH 6.5, 50 mM NaCl with 10 % D_2_O by repeated dilution/concentration.

### RNA production and purification

RNA samples were prepared by solid phase synthesis with standard 2′-*O*-TOM protected building blocks (ChemGenes). The 25 nt sequence 5′-GCUCUGACCGAAAGGCGUGAUGAGC-3′ was synthesized on an Applied Biosystems (ABI) 391 PCR Mate using an in-house written synthesis cycle. Custom primer support PS 200 (GE Healthcare) was used with an average loading of 80 µmol/g. Amidites (0.1 M) and activator (BTT, 0.3 M) solutions were dried overnight using freshly activated molecular sieves. The removal of protecting groups and cleavage from solid support were conducted by treatment with aqueous methylamine (40 %, 700 µl) and ammonia solution (33 % in water, 700 µl) at 40 °C for 90 min. After evaporation of the alkaline solvents, 2′-*O*-protecting groups were removed by dissolving the crude RNA in 1 M TBAF in THF (1.2 ml). After 14 h at 33 °C, the reaction was quenched by adding the same volume 1 M TEAA buffer (1.2 ml, pH 7.0, triethylammonium acetate). The volume of the solution was reduced to approximately 1 ml and applied to a 50 ml HiPrep 26/10 desalting column (GE Healthcare). The crude RNA was eluted with water, evaporated to dryness and re-dissolved in 1 ml of deionized water. The quality of the crude sequence was checked via anion exchange chromatography on a ThermoFisher DNAPac PA-100 column (4 × 250 mm). Purification was achieved by applying the crude RNA on a semipreparative ThermoFisher DNAPac PA-100 column (9 × 250 mm). The fractions containing the desired RNA were pooled, diluted with 0.1 M TEABC (triethylammonium bicarbonate) buffer and loaded onto a C18 SepPak cartridge (Waters). The RNA was eluted with acetonitrile/water (1:1) as the triethylammonium salt and lyophilized overnight.

### NMR spectroscopy

NMR experiments were carried out on 600 MHz Bruker Avance II+ and 500 MHz Agilent DirectDrive spectrometers at 25 °C. For backbone resonance assignment we used ^1^H-^15^N-HSQC, ^1^H-^13^C-HSQC, and HNCO, HN(CO)CA, HNCA, HNCACB, CBCA(CO)NH and ^15^N-HSQC-TOCSY (50 ms, 90 ms mixing time) triple resonance experiments. Data were processed using NMRPipe (Delaglio et al. 1995) and analyzed with CcpNmr (Vranken et al. 2005).

### Assignment and data deposition

**Fig. 1:**
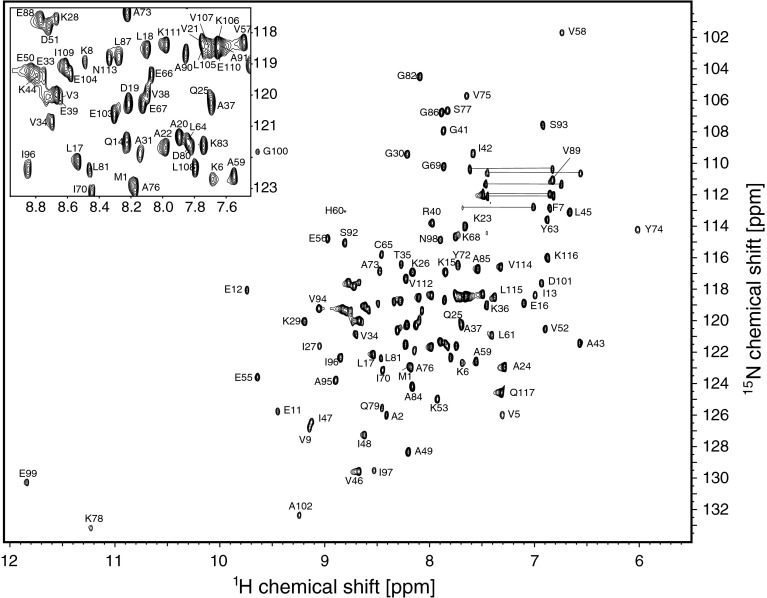
^1^H-^15^N-HSQC spectrum of ligand-free L7Ae (10 mM sodium cacodylate, pH 6.5, 50 mM NaCl, 10 % D_2_O, 25 °C). *Top left corner* zoom of center area of the spectrum. The position of residue H64 (below the intensity cutoff) is indicated by an *asterisk*. *Horizontal lines* represent NH_2_ side chain resonances

**Fig. 2:**
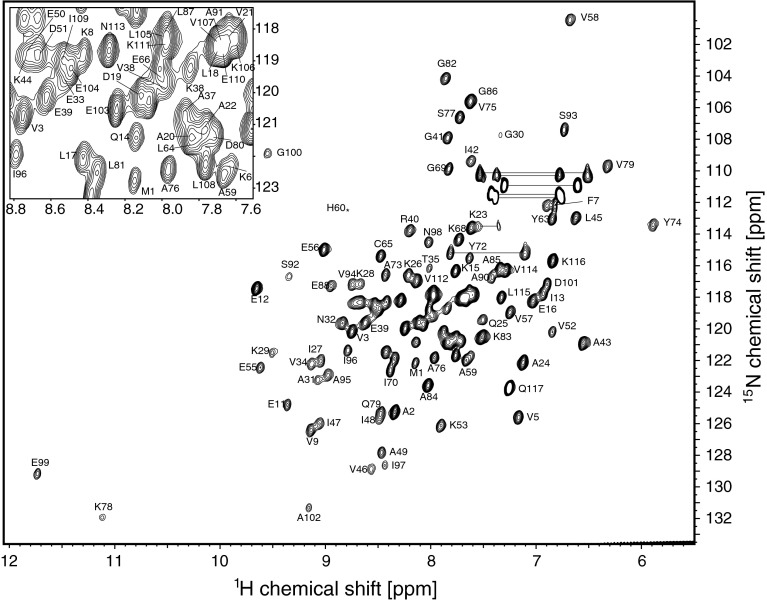
^1^H-^15^N-HSQC spectrum of L7Ae bound to the 25 nt RNA C/D box kink-turn (10 mM sodium cacodylate, pH 6.5, 50 mM NaCl, 10 % D_2_O, 25 °C). The position of residue H60 is indicated by an *asterisk*

Backbone amide ^1^H-^15^N resonance assignment of ligand-free L7Ae was achieved for 111 (of 113 non-proline) residues, corresponding to 98 % completeness (Fig. 1). Cα, Cβ resonances were assigned for all residues (100 %) and C′ assignments are 98 % complete. For RNA-bound L7Ae backbone amide ^1^H-^15^N resonance assignment was obtained for 111 (98 %) residues, while Cα, Cβ and C′ assignments were obtained for 99 %, 91 % and 98 %, respectively. The assigned ^1^H-^15^N-HSQC spectrum of the complex formed by L7Ae and the 25 nt RNA ligand is shown in Fig. 2. Complex formation is accompanied by a significant increase of the resonance linewidths.

**Fig. 3:**
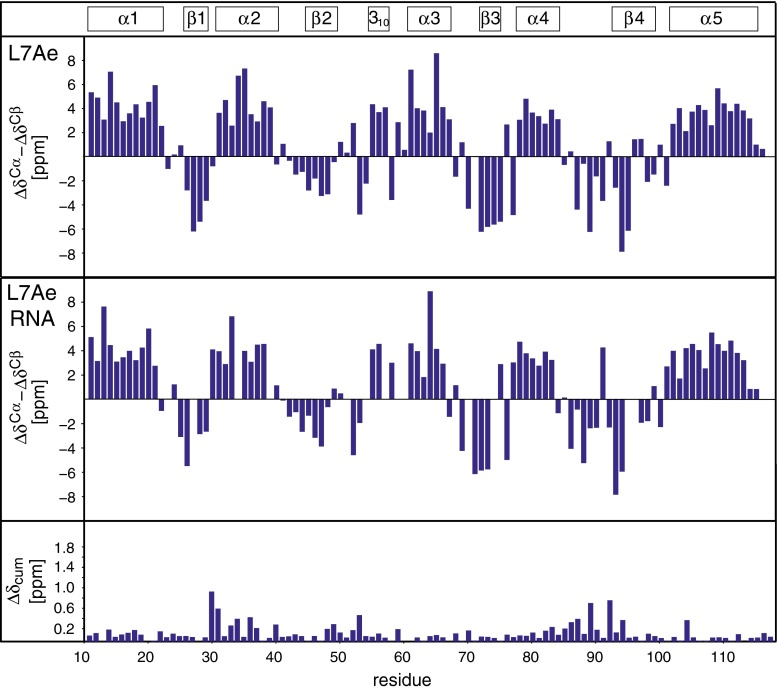
Difference of Cα and Cβ secondary chemical shifts (Δδ^Cα^ − Δδ^Cβ^) for ligand-free (*top*) and RNA-bound (*middle*) L7Ae (Tamiola et al. 2010). *Bottom* cumulative change in L7Ae backbone HN, N, Cα, Cβ and C′ chemical shifts (Δδ_cum_) upon RNA binding (Korzhnev et al. 2012). The secondary structure of L7Ae, as defined by Suryadi et al. (2005), is indicated

**Fig. 4:**
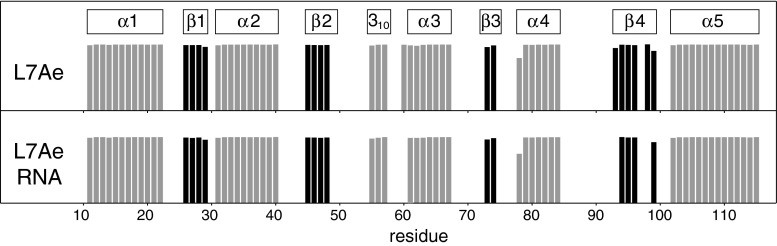
TALOS+ secondary structure prediction (*grey*: a-helix, *black*: b-sheet) of ligand-free (*top*) and RNA-bound (*bottom*) L7Ae, using backbone HN, N, Ca, Cb and C′ chemical shifts. The secondary structure of L7Ae, as defined by Suryadi et al. (2005), is indicated

The NMR secondary chemical shifts (Tamiola et al. 2010) for ligand-free and RNA-bound L7Ae are consistent with the crystal structures (Fig. 3). Using the L7Ae backbone HN, N, Cα, Cβ and C′ chemical shifts, a TALOS+ prediction of the secondary structures of both forms of the protein was performed (Shen et al. 2009). Overall, the secondary structures that were identified by TALOS+ are in very good agreement with the crystal structures of L7Ae (Fig. 4). Significant differences between the ligand-free and RNA-bound protein are not evident from the NMR chemical shift data, and only small cumulative changes in backbone HN, N, Cα, Cβ and C′ chemical shifts (Korzhnev et al. 2012) upon RNA binding are found (Fig. 3).

The protein chemical shift assignments of ligand-free and RNA-bound L7Ae and have been deposited at the Biological Magnetic Resonance Data Bank (http:www.bmrb.wisc.edu) with BMRB accession numbers 19907 (L7Ae) and 19908 (L7Ae-RNA complex).
